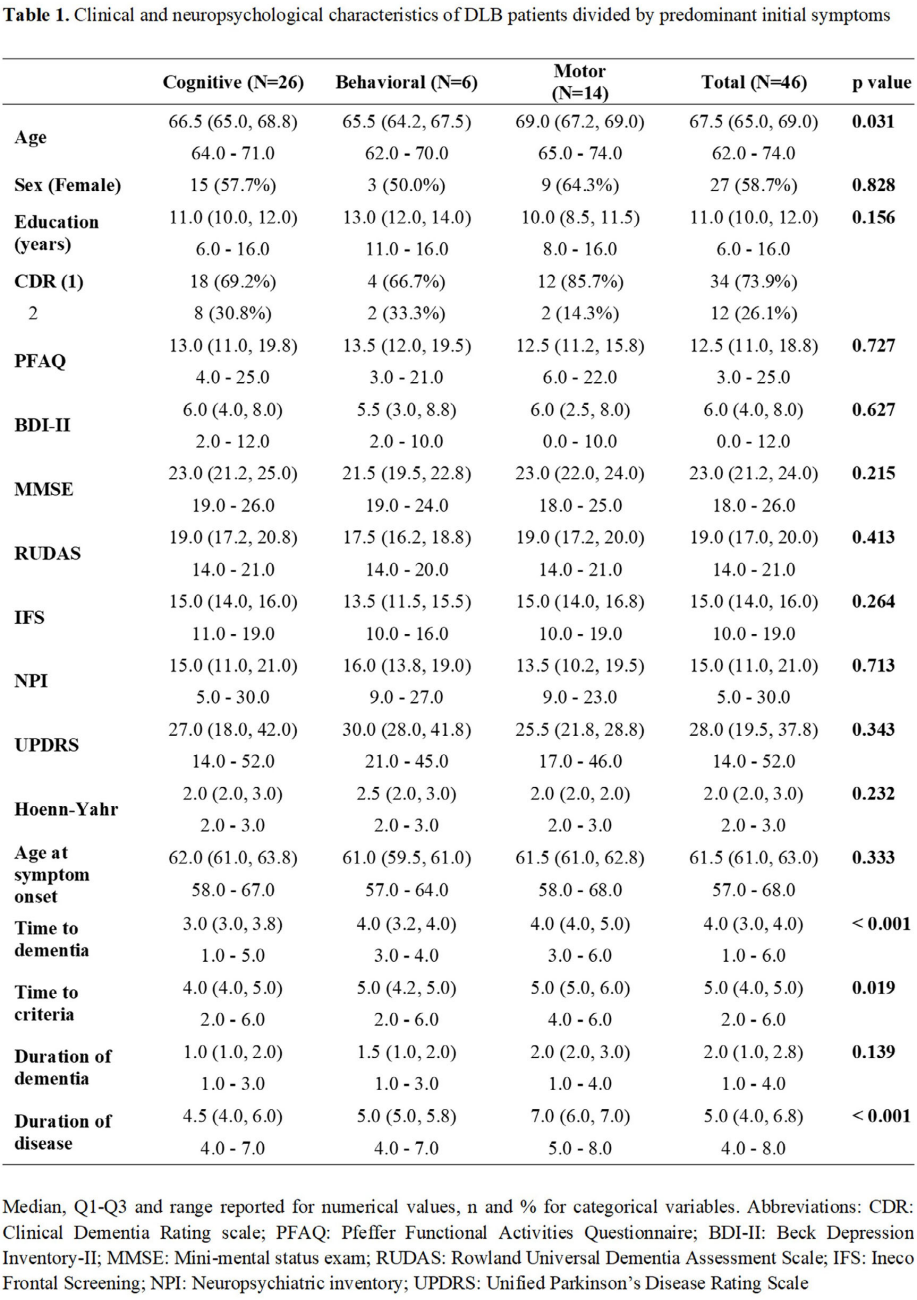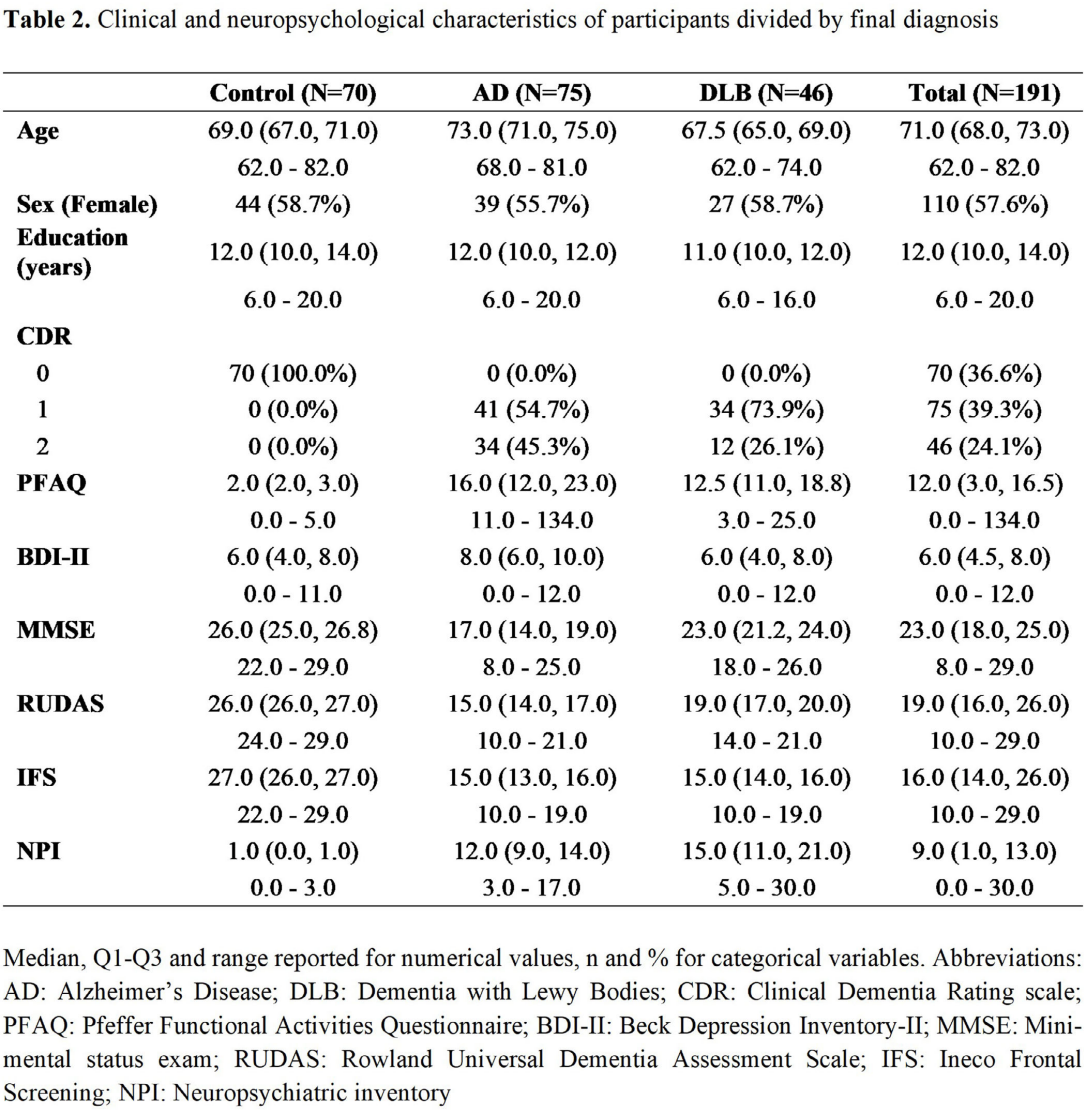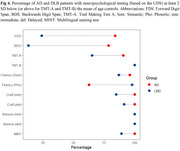# Clinical characterization of probable Dementia with Lewy Bodies based on the initial clinical presentation in a Latin American Low‐ and Middle‐ Income Country

**DOI:** 10.1002/alz70857_099298

**Published:** 2025-12-24

**Authors:** Nilton Custodio, Marco Malaga, Diego Bustamante‐Paytan, José Carlos Huilca, Katherine Aguero, Graciet Verastegui, Pamela Bartolo, Daniela Bendezu, Zadith Yauri, Rosa Montesinos

**Affiliations:** ^1^ Instituto Peruano de Neurociencias, Lima, Lima, Peru; ^2^ Unidad de Investigación de Deterioro Cognitivo y Prevención de Demencia, Instituto Peruano de Neurociencias, Lima, Peru; ^3^ Unidad de Investigación y Docencia, Equilibria, Lima, Peru, Lima, Peru; ^4^ Equilibria, Lima, Lima, Peru; ^5^ Unidad de Investigación y Docencia, Equilibria, Lima, Peru; ^6^ Universidad de San Martín de Porres, Facultad de Medicina, Centro de Investigación del Envejecimiento, Lima, Lima, Peru

## Abstract

**Background:**

Dementia with Lewy Bodies (DLB) is a specific type of dementia associated with the accumulation of alpha‐synuclein protein in the brain. The disease often presents heterogeneously in its early stages, and little is known about how this variability affects its progression. We investigated the initial clinical presentation of DLB among Peruvian patients and its impact on the clinical course.

**Method:**

We conducted a retrospective study of patients with DLB and Alzheimer's disease (AD). Participants were diagnosed using established clinical criteria. Neuropsychological assessments, including the MMSE, IFS, and UDS, were administered to evaluate cognitive function. We compared the clinical characteristics and neuropsychological profiles of DLB patients based on their initial presenting symptoms (hallucinations or parkinsonism). Statistical analyses, including ANOVA, chi‐squared tests, Wilcoxon tests, and multinomial linear regression, were performed to identify differences between groups and to investigate the impact of initial symptoms on disease progression and cognitive decline.

**Result:**

A total of 46 patients with probable DLB were included in the study. The median time from symptom onset to DLB diagnosis was approximately five years. Cognitive symptoms were the most common initial presentation, followed by motor and behavioral symptoms. However, no significant differences were observed in clinical or neuropsychological characteristics between groups at the time of evaluation. Compared to AD patients, DLB patients scored higher on measures of cognitive function (MMSE, RUDAS) and behavioral symptoms (NPI). Neuropsychological testing revealed distinct profiles between the two groups, with DLB patients demonstrating greater impairment in visuospatial and executive functions.

**Conclusion:**

DLB patients with initial cognitive symptoms developed dementia earlier compared to those with initial motor or behavioral symptoms. However, the initial presenting symptoms did not result in worse severity across specific cognitive or behavioral domains.